# Should we treat aging as a disease? The consequences and dangers of miscategorisation

**DOI:** 10.3389/fgene.2015.00171

**Published:** 2015-07-14

**Authors:** Richard G. A. Faragher

**Affiliations:** School of Pharmacy and Biomolecular Sciences, University of Brighton, Brighton, UK

**Keywords:** rapamycin, senescence, werner syndrome, history, ethics, medical, philosophy

## Abstract

The aging of the population represents one of the largest healthcare challenges facing the world today. The available scientific evidence shows that interventions are available now that can target fundamental “aging” processes or pathways. Sufficient economic evidence is available to argue convincingly that this approach will also save enormous sums of money which could then be deployed to solve other urgent global problems. However, as yet this scenario has barely entered the public consciousness and, far from being a point of vigorous debate, seems to be ignored by policy makers. Understanding why this lethargy exists is important given the urgent need to deal with the challenge represented by population aging. In this paper I hypothesize that one major cause of inaction is a widely held, but flawed, conceptual framework concerning the relationship between aging and disease that categorizes the former as “natural” and the latter as “abnormal.” This perspective is sufficient in itself to act as a disincentive to intervention by rendering those who hold it prone to the “naturalistic fallacy” but can give rise to active hostility to biogerontology if coupled with loose and/or blurred understanding of the goals and potential of the field.

## Introduction

### Terminology in a Contested Field

One problem when discussing the science of aging is that the same word often means different things to different people. In this article the word “aging” is used specifically to describe the operation of processes within whole organisms or populations of such organisms that result in an exponential increase in the chance of both death (mortality) and sickness (morbidity) with the passage of time (the Gompertz relationship). Again, in this article, “senescence” is restricted to cells from mitotic populations which have undergone irreversible exit from the cell cycle and display an altered phenotype. The accumulation of such senescent cells in the somatic tissues of organisms capable of replacing lost cells is one aging mechanism.

A related problem is a tendency for those discussing the “ethical issues” of aging research to conflate “immortality” and “living forever” with “extended lifespan” and both with being “non-aging.” This is unhelpful. Accordingly, the following definitions are used here. An “immortal” organism is one that will never die through any intrinsic cause. For the purposes of argument I assume it can still be killed by extrinsic means. It is important to note that no such organisms are currently known to exist. In contrast “non-aging” organisms do exist in the biosphere (*Arctica islandica* and *Hydra* being famous examples). Such organisms show a (relatively) fixed chance of sickness and death year on year in contrast to the exponential increase seen in species showing aging. Thus, with a chance of death that is fixed, but low, lifespans of centuries are achievable in the wild.

“Extended lifespan” is regularly used in the biogerontological literature with reference to mutations or interventions which cause individuals from a species which shows aging to live longer. To date, organisms in which lifespan has been extended have not been “converted” into non-aging organisms. Rather, the increase in their rates of mortality have been significantly attenuated, but not abolished, resulting in longer lifespans. References to “extended lifespan” tend to discount discussion of health status. Thus gerontologists prefer the term “healthspan” indicating an increase in the period of healthy life. Increased healthspan is a corollary of the interventions that produce extended lifespan but is not the same thing.

Lastly it should be noted that “lifespan” and “life expectancy” are not synonymous. Lifespan is the maximum length of time members of a given species can live. In contrast “life expectancy” is the likely survival time and is strongly influenced by environmental conditions. Thus in the 20th Century the life expectancy of humans increased dramatically (which is why there are more old people). In contrast lifespan appears to be unchanged (a maximum of ∼120 years).

### The Scale of the Challenge Posed by Aging

It is possible, but rare, to age in relatively good health. For the majority of people growing old is associated not just with an increased risk of death but also with a significant risk of developing a plethora of degenerative conditions and functional impairments Khaw (1999). This burden of morbidity is probably the most distressing aspect of old age and it has negative effects on two levels.

Firstly, morbidity incurs very significant costs on essentially every component of the health care system (Seshamani and Gray, 2004; Comas-Herrera et al., 2007; NHS National Statistics, 2008). At the level of the individual citizen, the simple truth is that growing old is a thoroughly miserable experience for an unacceptably large proportion of the population (Jylhä, 2004; Pérès et al., 2008). When rated against the standard benchmarks a biological gerontologist would use to measure successful aging (maintenance of normal function, avoidance of disease and social engagement) only about 18% of people can be described as undergoing “successful aging” Bowling and Dieppe (2005). When individuals are asked to self-rate whether they are aging successfully they draw on both physiological and psychosocial factors which results in larger apparent success scores. However, these are only of the order of 50–75% (Strawbridge et al., 2002). This difference illustrates two important points. The first is that it is possible to be happy with your lot against a background of morbidity. However, the second is that anywhere between half and a quarter of elderly people do not consider themselves to be aging well. They are not happy and are probably not healthy either.

Unless improvements occur, we may end up with a world in which we spend more money than ever before to keep more people than ever before more miserable than ever before. Improvement could come from two quarters. The first of these is the possibility that better primary prevention may postpone the age of onset of morbidity. If this occurs to a greater extent than life expectancy increases then the result would be a reduction in cumulative lifetime morbidity. This is a concept known as “compression of morbidity” first formally articulated by Fries (1980) who envisions it occurring by reducing the lifestyle risks to health which result in morbidity and disability. There is some evidence that this is occurring, at least in a limited form (Hessler et al., 2003; Crimmins, 2004; Fries, 2005).

The second option to improve population aging is to focus our efforts on understanding how the mechanisms driving aging operate. There is now evidence in model systems that individual aging mechanisms play a causal role in multiple morbidities (see below). Thus, it is plausible that interventions which simultaneously postpone multiple causes of morbidity could be developed and translated into practice. This focus on fundamental aging mechanisms, rather than on “age-related disease” represents a paradigm shift relative to previous ideas on the relationship between aging and disease.

### The Conceptual Relationship between Aging and Disease: Then and Now

Recognition that growing old is associated with ill health and death is at least as old as humanity. However, ideas from the world of classical antiquity are a good starting point for any discussion of how the relationship between physical decline and aging was historically conceptualized by physicians.

In this regard the Greco-Roman world is a better starting point than, for example, Han China because classical doctors exercised a marked influence over Western (and subsequently global) medical thinking until at least the 17^th^ century (Porter, 1999). The contribution of these early ideas to both clinical and societal conceptions of aging is thus so profound that they are worth examining at some length.

As Cockayne (2003) has shown, classical thinkers varied in their view of aging. The authors of the Hippocratic corpus (5th century BC) essentially regarded aging as a disease. This view was shared by Seneca (First century AD) and in a nuanced form by Aristotle (4th century BC) who saw aging as a “natural disease”-an interesting example of intellectual fence sitting. However, it was the greatest classical writer on medical matters, Claudius Galen (c130–c210 AD), who appears to have been the first to have explicitly considered aging as a “natural condition” standing in opposition to disease which he considered “contrary to nature.” A simple summary of this idea might thus be:

(1) Disease is defined as disordered or abnormal function.
(2) Aging is universal. Everyone “catches it.”
(3) That which is universal cannot be abnormal.
(4) Therefore, aging is not a disease. It is a “natural process.”

Galen’s view of a distinction between aging and disease was transmitted through the classical corpus and thus into modern societal conceptions of growing old. His ideas were directly echoed by highly influential modern medical thinkers including Aldred Warthin (1866–1931) “the father of cancer genetics” and Ludwig Aschoff (1862–1942) “the Nestor of arteriosclerosis research” (Achenbaum, 1995).

Within gerontology, Vladimir Korenchevsky (1880–1959) was perhaps the most notable proponent of this “Galenic” view. His early experiences as a clinician in Moscow convinced him that the disabilities he saw in the elderly were the result of disease rather than aging itself. According to Korenchevsky (1961) “old age is an abnormal, pathological syndrome, in which physiological processes of ageing are complicated and aggravated by various so-called degenerative diseases of old age.” His views were captured in an influential posthumous work *Pathological and physiological ageing.*

Korenchevsky’s view of aging is important not least because he was the founder of both the British Society for Research on Ageing and the International Association of Gerontology (IAG). Thus it is perhaps unsurprisingly that at the first meeting of this organization (Liege, Belgium 1950) the Honorary President identified the two major challenges before the participants as (1) the definition of aging and (2) the distinction between the effects of aging and disease. To require that such a distinction be drawn presupposes that the individual asserting this need already considers aging and disease to be separate entities. Although in 1957 the President of the Fourth Congress, Professor Greppi, dissented from this view and argued that aging should be viewed as a progressive disease terminating in death, a conceptual distinction between aging and disease was a general feature of thinking among IAG participants (Shock and Baker, 1988).

A particularly influential and articulate proponent of the aging-disease distinction was Nathan Shock (1906–1989). Shock was an early member of the Anglo-American “Club for aging” founded by Vladimir Korenchevsky (in 1949 he served as the secretary of the “American Branch” of the Club which would eventually become the Gerontological Society of America) and a significant figure within the IAG. Crucially, Shock was also one of the founders of the Baltimore Longitudinal Study on Aging (BLSA). The conceptual framework underlying this study was made clear by Shock et al. (1984) in *Normal Human Aging* in phrases of which Galen might have been proud.

“Although the incidence of disease increases with age, ageing and disease are not synonymous. Aging is a normal concomitant of the passage of time that takes place in everyone; disease occurs in only a part of the population…Although there are wide individual differences in the rate at which age changes take place ageing affects all members of a population, whilst specific diseases and accidents are selective” (Shock et al., 1984)

Hopefully, it is clear from the preceding summary that the accusation by De Grey and Rae (2007) that early gerontologists deliberately invented the distinction between ageing and disease because “by ring fencing their area of work intellectually, gerontologists hoped to ring-fence it financially” is unfounded and unfair. These early researchers were not making some cynical bid for a separate pot of grant money. Instead, they were echoing a medical tradition about the relationship between ageing and disease which predated not just the scientific method, but the English language.

It would also be mistaken to assume that researchers operating within a paradigm that distinguished between aging and disease considered “natural changes” to be benign. Their view has been eloquently summarized by Rowe et al. (1990).

“Even if one finds a change with age in carefully screened “normal” subjects, it is important to understand that normality does not necessarily mean harmlessness…although systolic blood pressure increases “normally” with age that does not mean it is harmless…Just because one defines some age-related changes as normative, one must not overlook their potential adverse effects”

Perhaps unfortunately for all concerned, this conceptual distinction between “natural” (and normal) aging and “unnatural” disease is ripe with the potential for fundamental philosophical error and “moral concern.” At the inception of the field this was of limited importance because the potential for clinical intervention in later life problems was very limited. However, this has changed.

### Modern Insights and Solutions from the Biology of Aging

In retrospect, the publication of *Normal Human Aging* occurred at a gerontological watershed. The mid 1980s could be said to be a period in which something was known about *why* aging occurred much was known about *what* changed as humans aged, but almost nothing about *how* this happened.

With the publication of Comfort’s, (1956) *Biology of senescence* it was recognized that aging is very common among species in the biosphere and thus provide a selective advantage to organisms which show it. A compelling explanation for the evolution of aging is based on the observation that the force of natural selection declines with age due to population attrition. As a result, even though the reproductive ability of “old” and “new” non-aging organisms are identical, under normal circumstances “old” organisms contribute fewer offspring to the next generation than the “new” organisms simply because there are fewer of them (Williams, 1957). Thus, any mutation that favors early life fecundity will be selected for even if it results in deleterious effects later on in the lifetime (a type of gene action termed *antagonistic pleiotropy*). Crucially, as well as explaining why aging happens, antagonistic pleiotropy also allows for the evolution of non-aging organisms provided they either lack a germ-line soma distinction (exemplified by the cnidarian *hydra*) or if the efficiency with which the organism produces offspring per unit energy increases over time.

Since aging evolved as the result of nothing more that the unprogrammed result of selection for early reproductive success, there is no requirement that the processes controlling the duration of life should be either (i) few in number of (ii) common between organisms. However, in the mid-1980s it became clear that this situation is in fact the case. Mutants in the insulin/insulin-like growth factor signaling pathway (IIS mutants) have been shown to have significantly extended lifespans (Piper et al., 2008; Selman et al., 2008). These pathways converge on the TOR protein and treatment of mice with the semi-selective mTOR inhibitor rapamycin extends lifespan by ∼20%, and slows many functional changes associated with aging. Rapamycin is a compound in routine clinical use and provides a potent proof of principle that drugs targeting fundamental healthspan maintenance pathways could be used clinically. Indeed, a related compound everolimus (RAD001) is now being used in trials to improve immune function in the elderly. It is well tolerated and shows significant beneficial initial effects (Mannick et al., 2014).

In parallel with this work it is now clear that the finite capacity to replace lost cells plays a causal role in mammalian aging. Senescence is the permanent entry of individual cells into a viable, but non-dividing state, usually as the result of repeated cell division. The molecular pathways which trigger this process are complex but are now relatively well understood. Cell senescence can be observed *in vitro* in cells from a wide variety of different species and acts as an anti-cancer mechanism. Considered in these terms, senescence appears to be an example of antagonistic pleiotropy at the process level. In the early part of the organismal lifespan entry into the senescent state probably prevents the growth of tumors, thus contributing to organismal survival. However, cellular senescence is typically associated with the heavily upregulated secretion of proinflammatory factors and other changes which have the potential to produce degenerative effects (Coppé et al., 2008; Burton et al., 2009; Kipling et al., 2009). As a result of declining clearance rates with age senescent cells accumulate in multiple tissues with *in vivo* age in a variety of species (Kipling et al., 2004; Herbig et al., 2006). The best evidence that senescent cells play a causal role in aging is the recent observation that their ablation in a transgenic mode (Baker et al., 2011) improves multiple aspects of aging (including metabolic dysfunction and wheel running). Most recently it has been shown that interdiction of key nodes of the pro-survival gene expression networks upregulated in senescence (either pharmacologically or using siRNA) killed senescent cells, but not their proliferating or quiescent, counterparts. *In vivo* this resulted in extended healthspan. Since the production costs of these first generation “senolytics” are low (a generic form of dasatinib could be produced for as little as $4 for a daily dose) such treatments are likely to be cost-effective (Zhu et al., 2015).

Crucially, the same mechanisms cause both age-related diseases, and features of aging considered in the past to be “natural changes” (e.g., the accumulation of senescent cells in the skin contributes to wrinkling, a “natural change” and to cardiovascular disease, an “age-related disease”). If the distinction between aging and age-related disease is false then the practical consequences of maintaining that such a distinction exists could be severe.

### The Implications of a Conceptual Divide between Aging and Disease

The existence of a conceptual divide between aging and disease is practically problematic for two reasons.

**A. Naturalistic Fallacy or the “Nofruit arguments”** Galen’s argument that aging and disease are distinct is easy to grasp, coherent and compelling. But it is important to recognize that it is essentially just an exercise in logic resting upon the definition of “disease” as abnormal function. Thinking about aging and disease like this raises surprising conceptual barriers to intervention. To illustrate this, imagine a land (let us call it “Nofruit”) where everyone has scurvy. Following Galen’s logic, in Nofruit scurvy is considered by the population to be a “natural condition.” This categorization immediately raises a potential barrier to action that is illustrated by Achenbaum (1995) below:

“Like other historians of aging, I indicate that most present day researchers on aging do not consider “old age” to be a disease. No “magic bullet” retards senescence.”

One interpretation of Achenbaum’s thinking is:

“Diseases have “magic bullets” or cures. Most authorities think ageing is not a disease so it cannot, by definition, have a cure.”

Thinking like this is, in itself, a disincentive to research. In Nofruit the line of thinking would go.

Diseases have “magic bullets” or cures. Most authorities think scurvy is not a disease so it cannot, by definition, have a cure.

Thus, most Nofruit scientists wouldn’t even try to find a cure for scurvy even though orange juice represents about as cheap and effective a “magic bullet” as can be imagined. The “problem” of scurvy would be tacitly ignored, much the way the possibility of successful intervention in aging is tacitly ignored in the real world.

However, let us assume that in Nofruit a researcher or clinician has not been discouraged by Achenbaum’s argument and has discovered that scurvy is curable. Any such researcher wishing to provide a rationale for the treatment of scurvy will essentially recapitulate the argument of Rowe et al. (1990), normality does not mean harmlessness.

So far so good, but the conceptual problem with “treating” the “normal” is that any decision to do so may potentially fall foul of the “naturalistic fallacy.” This is the (erroneous) philosophical position that equates the normal (or natural) with the “good” and the “unnatural” with its converse. On this fallacious logic avalanches, volcanoes and tsunamis are “good” (because they are natural) whilst books, houses and clothing are bad (because they are not found in nature). When applied to aging (or to scurvy in Nofruit) the naturalistic fallacy leads some actors to conclude that aging (or scurvy) shouldn’t be “interfered with” (note use of the term, rather than “treated”). It is important to note that treating a “disease” does not trouble an actor in the grip of the naturalistic fallacy because diseases are conceptualized as “abnormal” or “unnatural” by definition and can accordingly be treated, because they are bad.

Not all treatments will necessarily trigger this response (in the real world, for example, male impotence is a common age-related change yet there is no serious effort to ban Viagra) but the potential for any particular intervention to do so is always present and acts as a further disincentive to treatment. The naturalistic fallacy and related arguments are one root cause of debates around “the medicalization of old-age” (Bury, 2005). In Nofruit a proportion of the population would worry about “the medicalization of scurvy.”

**B. False division of unity**. It is important to recognize that the Nofruit arguments do not require aging and age-related disease to share causal mechanisms. Both may cause harm in different ways. However, in actuality the mechanisms which cause aging and age-related disease really do overlap very substantially. Thus distinguishing between “aging” and “age-related disease” probably represents an artificial distinction; human understanding has drawn an arbitrary line on the complex phenotype which is later life.

Maintaining an artificial aging-disease distinction give rise to a contradiction. What is the ethical rationale for treating entities classified as “diseases” caused by senescent cells (like cardiovascular disease) but not treating entities classified as “natural changes” (like wrinkles) which are also caused by senescent cells? As yet this problem does not seem to have been fully recognized by bioethicists, probably because the science on which it is based is so new that it has not yet been disseminated. The little which has been said on the topic however, offers gerontologists little reassurance that our work will be well received.

### From Indifference to Moral Concern

Blackburn (2001) has pointed out “for human brings there is no living without standards of living.” Ethical frameworks determine the things we find acceptable, or admirable or contemptible. Put simply, they tell us, as actors, what is right and what is wrong. Thus, branding a course of action as immoral will, all things being equal, act as a significant disincentive to action. Good people do not willingly do bad things.

The prospect of successful intervention into the causal mechanisms of aging has produced moral concern most notably from the bioethicist Leon Kass who wrote in *L’Chaim and Its Limits: Why Not Immortality?* (Kass, 2001).

“Should we…welcome efforts to increase not just the average but also the maximum human life span, by conquering aging, decay, and ultimately mortality itself?”

Kass, takes the view that

“Confronted with the growing moral challenges posed by biomedical technology, let us resist the siren song of the conquest of aging and death.”

Appleyard (2007) in *How to live forever or die trying* is even more explicit in his opposition to biogerontology. He is careful first to define what he calls “medical immortality”:

“We shall not be immortal in the sense that we cannot die, plainly we can still be killed. But we could not be killed by disease or age our bodies would be immune to infection, dysfunction or the ravages of time. We would be medically immortal.”

After about 300 pages spent detailing his travels into the wilder fringes of biogerontology and life-extension Appleyard concludes:

“There is a deep and absolutely unavoidable selfishness involved in the idea of immortality. Billions have lived and died, why should I in particular be immortal? Why should I persist in being?”

Selfishness is bad (even if Appleyard’s version sounds more like survivor guilt). Therefore for Appleyard, since immortality requires deep selfishness, immortality is deeply bad. By implication any research which facilitates such a goal is bad. Biogerontology facilitates such a goal. Therefore biogerontology is bad.

Biogerontologists are probably well advised to take arguments of the type advanced by Kass and Appleyard seriously. This is because they may resonate with ideas which a proportion of the population in some countries share to some extent (Ipsos-MORI, 2006; Pew Research, 2013). Such views discourage policy makers from targeting aging mechanisms to treat the health problems seen in later life and at worst they suggest that gerontology itself is morally wrong. What response can gerontologists reasonably offer? I would suggest the following:

From what we currently know about the biology of aging the most that could be conceived as scientifically deliverable, even centuries from now, is to be “non-aging” (as defined in Terminology in a Contested Field). But non-aging organisms are not in a state remotely comparable to Appleyard’s “medical immortality” they have very long, but finite, lifespans and die from “inside” like aging organisms, just at much lower rates.

This point is crucial because both Appleyard and Kass (in different ways) treat immortality as a state humans could achieve. Medical immortality is then held up to ethical scrutiny and found wanting. But since “immortality” is a state that humans can never achieve all the authors are offering is, at bottom, a moral critique of never-never land. Immortality may be bad but biogerontology cannot be bad by association since it cannot help anyone become immortal. Neither can anything else.

One problem within speculative writing in this area is the sloppy tendency to equate “extended lifespans,” “1000 year lifespans” and “immortality.” Given that the Earth is about 4.5 billion years old, a human lifespan stretched to a millennium (or even ten millennia) represents little more than the blink of an eye and is not realistically on offer, any more than “medical immortality.”

However, it is noteworthy that a long, but finite, life is a different moral proposition from “immortality” unless one takes the view that humans have a “natural” lifespan, adherence to which is a moral good. But this line of thinking it is very close to the naturalistic fallacy with the added sting that (given what we now know about the shared mechanisms between “aging” and “age-related disease”) it renders treating age-related disease unethical (or perhaps treating any disease at all unethical since smallpox and syphilis are “natural” components of the biosphere).

In fairness to Appleyard and Kass they seem to have arrived at the conclusion that “1000 year lifespans” and “immortality” are possible in part because of utterances from figures on the scientific fringe. Of these fringe figures Aubrey De Grey is the best known and most articulate and his writings may thus stand for the whole. In *Ending Aging* De Grey summaries current progress in gerontology and writes:

“I expect many people alive today to live to one thousand years of age and to avoid age-related health problems even at that age.” Even more bullishly he sketches a vision of largely unimpeded progress (which he terms “longevity escape velocity”) and continues:

“There is a strong chance that you-the reader of this book-will live to experience the rejuvenation of your body leading ultimately to an endless summer of literally perpetual youth.”

De Grey is committing something of a logical fallacy. The existence of impressive scientific breakthroughs today does not pre-ordain impressive scientific breakthroughs tomorrow. If scientists start to talk like science fiction writers, where will the public go for scientists? Vain hopes beget vain fears.

Lastly, although there is doubtless some overlap between popular views about the validity of aging research and the thoughts of Appleyard and Kass it is less clear how much of their thinking is shared among the public. When discussing extended healthspan Kass comments:

“Some, of course, eschew any desire for longer life. They seek not adding years to life, but life to years… This has much to recommend it. Who would not want to avoid senility, crippling arthritis, the need for hearing aids and dentures, and the degrading dependencies of old age? But, in the absence of these degenerations…We could no longer comfort the widow by pointing out that her husband was delivered from his suffering.”

However, research into public attitudes to gerontological research in the UK indicated a desire among the participants for a long and active life rather than to serve as object lessons in deliverance from suffering. de Magalhães (2014) has suggested that the concerns shown about extended lifespans by some participants in the Pew Research survey may result from their belief that these would be associated with the kind of morbidity seen in aging Americans today. If so this reinforces the key message that healthspan is the outcome most desired by our populations. The most effective way to facilitate this would be to significantly increase the funding available for research into the fundamental biology of aging and facilitate the rapid translation of its discoveries into the clinical arena.

### Conflict of Interest Statement

The author declares that the research was conducted in the absence of any commercial or financial relationships that could be construed as a potential conflict of interest.
